# Multi-facetted impulsivity following nigral degeneration and dopamine replacement therapy

**DOI:** 10.1016/j.neuropharm.2016.05.013

**Published:** 2016-10

**Authors:** Michel Engeln, Solène Ansquer, Emilie Dugast, Erwan Bezard, David Belin, Pierre-Olivier Fernagut

**Affiliations:** a Univ. de Bordeaux, Institut des Maladies Neurodégénératives, UMR 5293, F-33000, Bordeaux, France; b CNRS, Institut des Maladies Neurodégénératives, UMR 5293, F-33000, Bordeaux, France; c Service de neurologie de l’hôpital de Poitiers, F-86021, Poitiers, France; d Université de Poitiers, F-86000, Poitiers, France; e CIC INSERM 1402, CHU de Poitiers, Poitiers, France; f INSERM U1084 Laboratoire de Neurosciences Experimentales et Cliniques, F-86000, Poitiers, France; g Department of Pharmacology, University of Cambridge, CB2 1PD, Cambridge, UK; h Behavioural and Clinical Neuroscience Institute of the University of Cambridge, CB2 3ED, Cambridge, UK

**Keywords:** Parkinson, Impulsivity, Alpha-synuclein, Pramipexole, Substantia nigra

## Abstract

Impulse control disorders (ICDs) are debilitating side effects of dopamine replacement therapy (DRT) in Parkinson’s disease (PD) that severely affect the quality of life of patients. While DRT, the pattern and extent of neurodegeneration, and prodromic factors of vulnerability (e.g. impulsivity) have all been hypothesized to play a role in the development of ICDs, their respective, and potentially interacting, contributions remain to be established. High impulsive (HI), Intermediate (Int) or low impulsive (LI) rats were identified based on their performance in both a differential reinforcement of low rate of responding (DRL) and a fixed consecutive number (FCN) schedules, that operationalize two independent facets of impulsivity, waiting and action inhibition (motor impulsivity). We investigated whether high impulsivity trait influenced the progressive development of a parkinsonian state induced by viral-mediated overexpression of α-synuclein, and whether impulsivity trait and nigrostriatal neurodegeneration independently or jointly influenced the effects of DRT on impulse control. α-synuclein-induced nigrostriatal neurodegeneration increased both waiting and motor impulsivity. The D2/D3 dopamine receptor agonist pramipexole exacerbated motor impulsivity more than waiting. However, the pramipexole-induced increase in waiting impulsivity observed in both sham and lesioned rats, was more pronounced in HI lesioned rats, which displayed a restricted α-synuclein-induced dopaminergic neurodegeneration. Thus, a PD-like nigrostriatal lesion increases both motor and waiting impulsivity, but its interaction with a pre-existing impulsivity trait, which, at the cellular level, confers resilience to dopaminergic neurodegeneration, worsens the detrimental effects of D2/D3 dopamine receptor agonists on inhibitory control.

## Highlights

• Degeneration of the substantia nigra increases both waiting and action impulsivity.
• Reduced alpha-synuclein-induced degeneration in high impulsive rats.
• Pramipexole increases waiting and action impulsivity in sham and lesioned rats.
• Exacerbated effect of pramipexole in high impulsive vs. low impulsive lesioned rats.

## 1 Introduction

Parkinson’s disease (PD) is a neurodegenerative disorder characterized both by degeneration of several neuronal populations, including dopaminergic neurons in the substantia nigra *pars compacta* (SNc), and presence of Lewy bodies, the anatomo-pathological hallmark containing aggregated α-synuclein. Dopamine replacement therapy (DRT) is the first-line treatment for alleviating the motor symptoms of PD but triggers impulse control disorders (ICDs) in vulnerable individuals (Weintraub et al., 2010). ICDs include behaviors such as pathological gambling, hypersexuality, binge eating or compulsive shopping, which impinge on the quality of life of the patients (Voon et al., 2009). The factors contributing to the development of these debilitating side effects remain poorly known. If dopamine overdose may account for some of the deleterious effects of DRT on cognitive processes, dopaminergic depletion and DRT by definition co-exist in all PD patients yet ICDs are only developed by a subset of them (Weintraub et al., 2010), suggesting a role for other factors, such as impulsivity. Indeed, contrasting with the parkinsonian personality usually described as rigid, introverted and slow tempered (Todes and Lees, 1985), novelty seeking, hypomania/extraverted personality, but also impulsivity have been linked to these compulsive behaviors in PD (Dagher and Robbins, 2009, Voon and Fox, 2007, Voon et al., 2011b). PD patients with ICDs display impairments in risk evaluation (Voon et al., 2011a), learning from outcomes (Piray et al., 2014, Voon et al., 2010a) and increased impulsivity (Voon et al., 2010b), alongside alterations of fronto-striatal and cortico-subcortical networks (for review (Tang and Strafella, 2012)).

Impulsivity is a multifaceted construct involving aspects of action inhibition (motor impulsivity) and waiting, both contributing to an inability to withhold prepotent, inappropriate, premature, responses (D’Amour-Horvat and Leyton, 2014, Dalley et al., 2008, Dalley and Roiser, 2012). Motor and waiting impulsivity are related to a dysfunctional dopaminergic modulation of corticostriatal networks (Antonelli et al., 2014, Basar et al., 2010, D’Amour-Horvat and Leyton, 2014, Jentsch et al., 2014): while drugs enhancing dopamine transmission increase, and dopamine antagonists decrease, motor impulsivity, the opposite modulation is found for waiting impulsivity (reviewed in (D’Amour-Horvat and Leyton, 2014, Dalley et al., 2008, Dalley and Roiser, 2012)). Thus, DRT increasing dopamine signaling may exacerbate motor impulsivity, while dopaminergic cell-loss would decrease dopamine levels and increase waiting impulsivity. Such influences of pharmacological manipulations of dopamine transmission may be biased in PD by the asymmetry in denervation between the relatively spared mesocorticolimbic network and the severely damaged nigrostriatal pathway. Consequently, DRT may induce a dopaminergic overdose of the nucleus accumbens and frontal cortex that may increase impulsivity (Cools et al., 2003, Gotham et al., 1988) in a state-dependent manner (Caprioli et al., 2013).

We hypothesized that baseline individual differences in impulse control may influence the effects of DRT on impulse control after nigrostriatal degeneration. To this end, we performed a longitudinal study investigating the respective, and interacting, contributions of premorbid impulsivity trait, progressive nigrostriatal dopaminergic neurodegeneration and DRT to the development of impulse control deficits in rats with viral-mediated overexpression of α-synuclein. We measured the influence of the bilateral nigrostriatal lesion and DRT on inhibitory control of rats displaying high or low levels of motor and waiting impulsivity as assessed by fixed consecutive number (FCN) and differential reinforcement of low rate of responding (DRL) schedules (Jentsch et al., 2014, Rivalan et al., 2007).

## 2 Materials and methods

### 2.1 Subjects

Forty-four male Sprague Dawley rats (Janvier, France, 200–225 g at the beginning of the experiment) were housed in pairs on a reversed 12 h cycle. After 5 days of habituation, they were food restricted to 90% of their free feeding weight during behavioral testing. Water was available *ad libitum*. Experiments were approved by the Institutional Animal Care and Use Committee of Bordeaux (CE50, license # 5012099-A) and performed under the European Union directive (2010/63/EU) on the protection of animals used for scientific purposes.

### 2.2 Behavioral procedures

Rats were challenged in two different tasks to assess individual ability both to inhibit prepotent responses, or wait (DRL), and maintain ongoing responses (FCN). In the DRL schedule, rats must wait for a specific time prior to responding on a manipulandum to obtain a reward: they have to inhibit a prepotent response. In the FCN schedule, rats must maintain a response on a first manipulandum for a fixed number of times before responding on a second one to obtain a reward. They therefore must not interrupt an ongoing instrumental response. Inabilities to wait in the DRL or to maintain the ongoing response chain in the FCN represent waiting and action impulsivity.

The sequences of training for each task were counter-balanced to avoid any carry-over effect. Results from preliminary experiments demonstrated that the acquisition and performance in the DRL and FCN tasks were not influenced by the nature of the instrumental response. We therefore used lever presses and nose-pokes as instrumental responses in the DRL and FCN task, respectively. Experiments were performed in operant chambers (DRL: 29.5 × 32.5 × 23.5 cm; FCN: 24 × 24 × 26 cm, Med Associates, USA) enclosed in sound-attenuating ventilated cubicles. Before being randomly assigned to a FCN-DRL or DRL-FCN group (See Suppl Fig. 1 for experimental design), rats were subjected to one session of magazine training, followed by three successive fixed-ratio 1 schedule sessions (100 lever presses in 45 min) prior to DRL and FCN training (45-min daily sessions).

#### 2.2.1 Differential reinforcement of low rate of responding 20s (DRL-20s)

Operant chambers were equipped with two levers located on the right and left side of a food tray. Above each lever was a white cue light, and a white house-light was on the opposite wall. The procedure was adapted from (Fletcher, 1995). Rats obtained a pellet if at least 5s had elapsed since their previous response on the reinforced lever (DRL-5s). When the subject reached >80% of rewards, the behavioral requirement was incremented to DRL-10s, -15s and -20s. All rats were tested under DRL-20s for 15 days before surgery. Premature responses reset the “waiting period” and were not rewarded. The first response was always reinforced. The following parameters were recorded: number of responses on the active/inactive levers, number of earned reinforcers, efficiency ([number of reinforcers/number of responses] × 100), inter-response time, number of food tray nosepokes, reward collection latency and seeking behavior (number of visits to the magazine/number of earned reinforcers).

#### 2.2.2 Fixed consecutive number schedules 16 (FCN16)

Operant chambers were equipped with a house-light and 2 holes in which rats could make nosepokes at the right and left side of a food tray located on the front wall. The procedure was adapted from (Evenden, 1998, Rivalan et al., 2007). Rats had to respond at least once (FCN1), three (FCN3), six (FCN6), eight (FCN8), twelve (FCN12) or sixteen (FCN16) times on the chain poke followed by a response in the reinforced hole to earn a pellet. Rats reached the following stage if they performed >80% successful trials during the session. Rats were tested under FCN16 over 15 days before surgery. The following parameters were recorded: number of responses on chain/reinforcement pokes, number of earned pellets, number of chains (number of responses on the chain poke before responding on the reinforcement poke), efficiency ([number of reinforcement poke/number of reward earned] × 100) and average chain length.

#### 2.2.3 Control experiment: effects of bilateral SNc lesion on the acquisition of a new task, operant responding and motivation

To exclude any potential confounding effect of lesion-induced impairments on general motivation or motor/instrumental performance, rats were trained in a different instrumental task (wheel-turning) under both continuous (FR1) and progressive ratio (PR) schedules of reinforcement. Operant chambers were equipped with one wheel and one 4 cm wide lever installed, respectively, at the left side and the right side of the back wall, and a food tray at the center of the front wall. Above the wheel was a white cue light. Twelve weeks after surgery, animals were subjected to 7 sessions of Fixed Ratio (FR1) schedule of reinforcement in which they had to perform 1/2 wheel turn to earn one reward (maximum 100 rewards or 60 min). Subsequently, rats were subjected to a progressive ratio (PR) of 1–100 pellets maximum to assess their motivation. The session ended if 15 min had elapsed without reinforcement.

### 2.3 Viral-mediated lesion

Under isoflurane anesthesia, rats were placed in a stereotaxic frame (Kopf, USA) and received two bilateral injections in the SNc (Anteroposterior: −5.1 and −5.4; Mediolateral: ±2.2 and ± 2; Dorsoventral: −7.8, in mm from bregma) of AAV2-9 expressing human A53T mutant alpha-synuclein driven by the synapsin-I promoter (n = 23) or AAV2-9 expressing the green fluorescent protein (GFP; n = 21) as previously described (Bourdenx et al., 2015, Engeln et al., 2013). Forelimb akinesia was assessed with the stepping test (Bourdenx et al., 2015, Engeln et al., 2013, Engeln et al., 2014) before and after (4, 8 and 12 weeks) surgery and after drug challenges.

### 2.4 Drugs

Levodopa (12 mg/kg + 15 mg/kg benserazide; i.p. 30 min prior to tests), apomorphine (0.1 mg/kg; s.c. 15 min prior to tests) and pramipexole (PPX, 0.3 and 1 mg/kg; i.p. 30 min prior to tests) were dissolved in 0.9% NaCl (drugs from Sequoia Research Products, UK excepted apomorphine, Aguettant, France). Doses were chosen as being efficient to improve motor deficits, as demonstrated with stepping test performances and previously shown (Engeln et al., 2013, Rokosik and Napier, 2012). All rats were tested under each drug condition using a Latin square design separated by washout sessions.

### 2.5 Histology

Rats were euthanized with a lethal injection of chloral hydrate (600 mg/kg; VWR, France) and perfused with 0.9% NaCl followed by 4% paraformaldehyde (PFA). After 24 h post-fixation in 4% PFA, brains were cryoprotected in PBS/20% sucrose then frozen. 40 μm-thick free-floating coronal sections were collected throughout the mesencephalon. Immunohistochemistry against tyrosine hydroxylase (TH, Millipore, France) and α-synuclein (Thermoscientific, UK) was followed by cresyl violet counterstaining. Stereological estimation of the total number of TH-positive neurons was performed with the optical fractionator method (Mercator, Explora Nova, France) (Bourdenx et al., 2015, Engeln et al., 2012, Engeln et al., 2013).

### 2.6 Data and statistical analyses

Data are expressed as mean ± SEM. HI (n = 11), Intermediate (n = 23) and LI rats (n = 10) were segregated by a K-means cluster analysis as previously described (Murray et al., 2015), carried-out on the averaged performance of baseline sessions 46/47 and 45/46 under FCN and DRL schedules, respectively. These sessions represent the last two of a series of 10 sessions of stable performance under the final pre-surgery stage (DRL-20s and FCN16). HI, Int and LI rats were then randomly allocated to the sham or lesioned group. Analysis of baseline data revealed no significant correlation between FCN and DRL performances (R^2^ = 0.018, p > 0.05), indicating that these two tasks reflect, as predicted, independent constructs pertaining to impulse control.

Behavioral differences between groups were analyzed with multifactorial, repeated measures (RM) analysis of variance (ANOVA), when appropriate. Assumptions about normality of distribution and homogeneity of variance were assessed using the Kolmogorov-Smirnov and Levene tests, respectively. Distributions were analyzed using chi-squared tests and correlations were tested using linear regressions. Stereological counts were analyzed using Student unpaired *t*-test. The effect of impulsivity on neuronal counts was analyzed with a two-way ANOVA with lesion and impulsivity as between-subject factors. Upon significant main effects, post hoc analyses were performed using the Newman-Keuls test. Statistical significance was accepted at p < 0.05.

## 3 Results

### 3.1 α-Synuclein-induced motor deficits and dopaminergic neurodegeneration

Baseline performances under differential reinforcement of low rate of responding 20s (DRL-20s, waiting impulsivity) and fixed consecutive number schedules 16 (FCN16, impulsive action) were used to segregate low impulsive (LI), intermediate (Int) and high impulsive (HI) subpopulations. Rats were then randomly allocated to the sham or lesioned group (See Suppl Fig. 1 for experimental design).

**Fig. 1:**
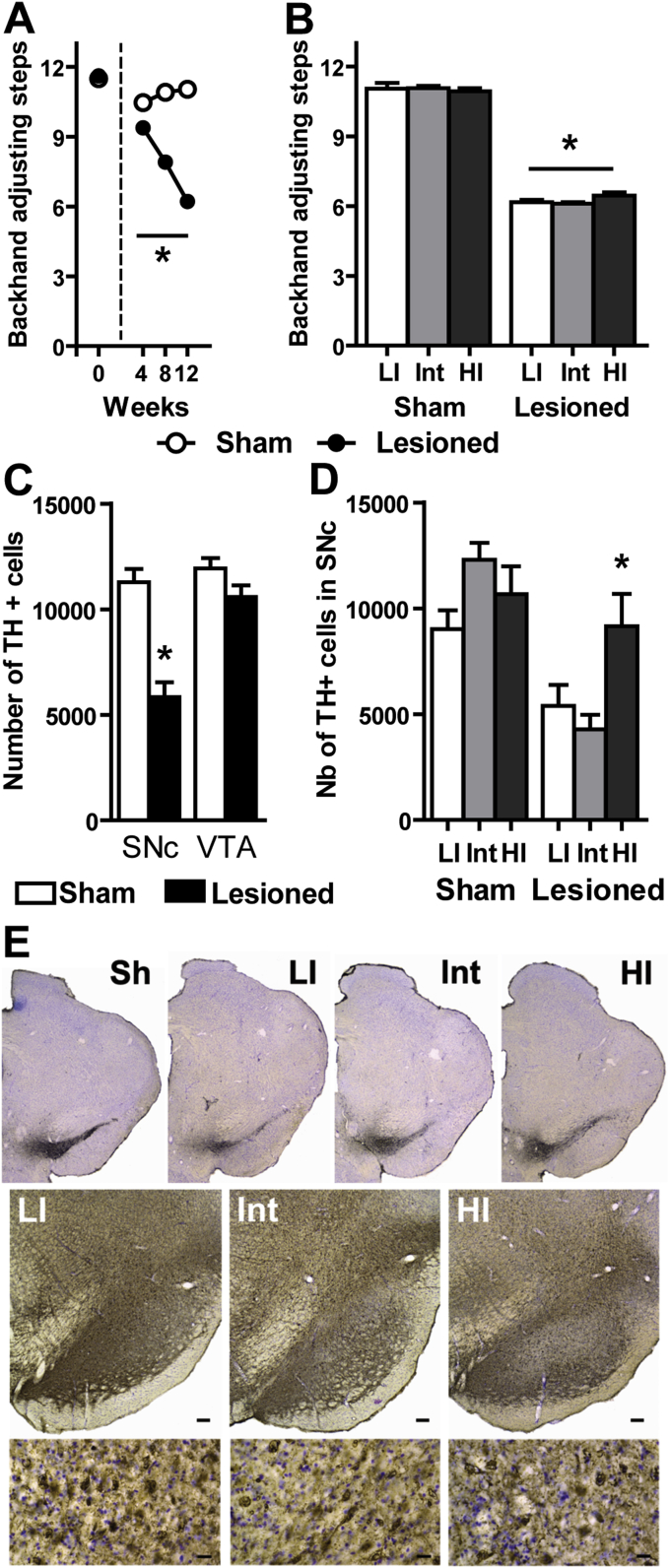
*α-Synuclein-induced motor deficits and dopaminergic neurodegeneration*. **A**. Stepping test performances at Baseline (0), 4, 8 and 12 weeks after viral-mediated expression of α-synuclein in the substantia nigra (p < 0.001 for each time-point; 2-way RM-ANOVA); **B**. Motor performances at 12 weeks post-surgery for the 3 subgroups of each population (*p < 0.001 from sham; 2-way ANOVA); **C**. Stereological counts of tyrosine hydroxylase (TH) positive cells in the Substantia nigra *pars compacta* (SNc) and the ventral tegmental area (VTA), *p < 0.0001 (*t*-test; all impulsivity traits included); **D**. Magnitude of the nigral lesion in Low impulsive (LI), Intermediate (Int) and High impulsive (HI) animals (*p < 0.05 from Lesioned LI and Int; 2-way ANOVA); **F**. Representative mesencephalic sections of one sham rat and lesioned rats of each impulsivity subgroup (upper panel) stained for TH. Low magnification pictures of α-synuclein immunostaining in each subgroup (middle panel; scale bar: 100 μm). High magnification images of the corresponding LI, Int and HI animals (lower panel; scale bar: 20 μm) showing α-synuclein accumulation in all subgroups. Sh = sham; LI = low impulsive; Int = intermediate; HI = high impulsive. Data represent mean ± SEM. Sham: LI: n = 5, Int: n = 12, HI: n = 4; Lesioned: LI: n = 6, Int: n = 11, HI: n = 6.

In all rats, viral-mediated expression of α-synuclein in the SNc elicited progressive bilateral motor deficits (Lesion × Weeks: F_3, 126_ = 365.7; p < 0.0001) significant at 4 weeks post-surgery, and worsening at 8 and 12 weeks (p < 0.001, Fig. 1A). 12 weeks post-surgery, HI, Int and LI lesioned rats displayed similar motor deficits (Fig. 1B) that were improved by all dopaminergic drug tested (each drug was tested on representative samples of 5–8 animals containing LI, Int and HI rats; Suppl Fig. 2).

Stereological counts revealed that overexpression of α-synuclein resulted in an overall 49% lesion restricted to the SNc (t = 5.79, df = 42; p < 0.0001; Fig. 1C). The extent of the lesion was dependent upon pre-existing impulsivity (Lesion × Impulsivity: F_2, 38_ = 6.01; p < 0.01) and HI rats displayed a milder lesion (19%, non significantly different from sham animals) than Int (62%) or LI (52%) (p < 0.05; Fig. 1D, E). This difference was specific to impulsivity trait as it was attributable neither to off-target injections of the viral vector nor to a lack of α-synuclein expression (Fig. 1E), as revealed by prominent α-synuclein immunoreactivity in the SNc of all rats.

### 3.2 Effect of dopaminergic neurodegeneration on inhibitory control

**Fig. 2:**
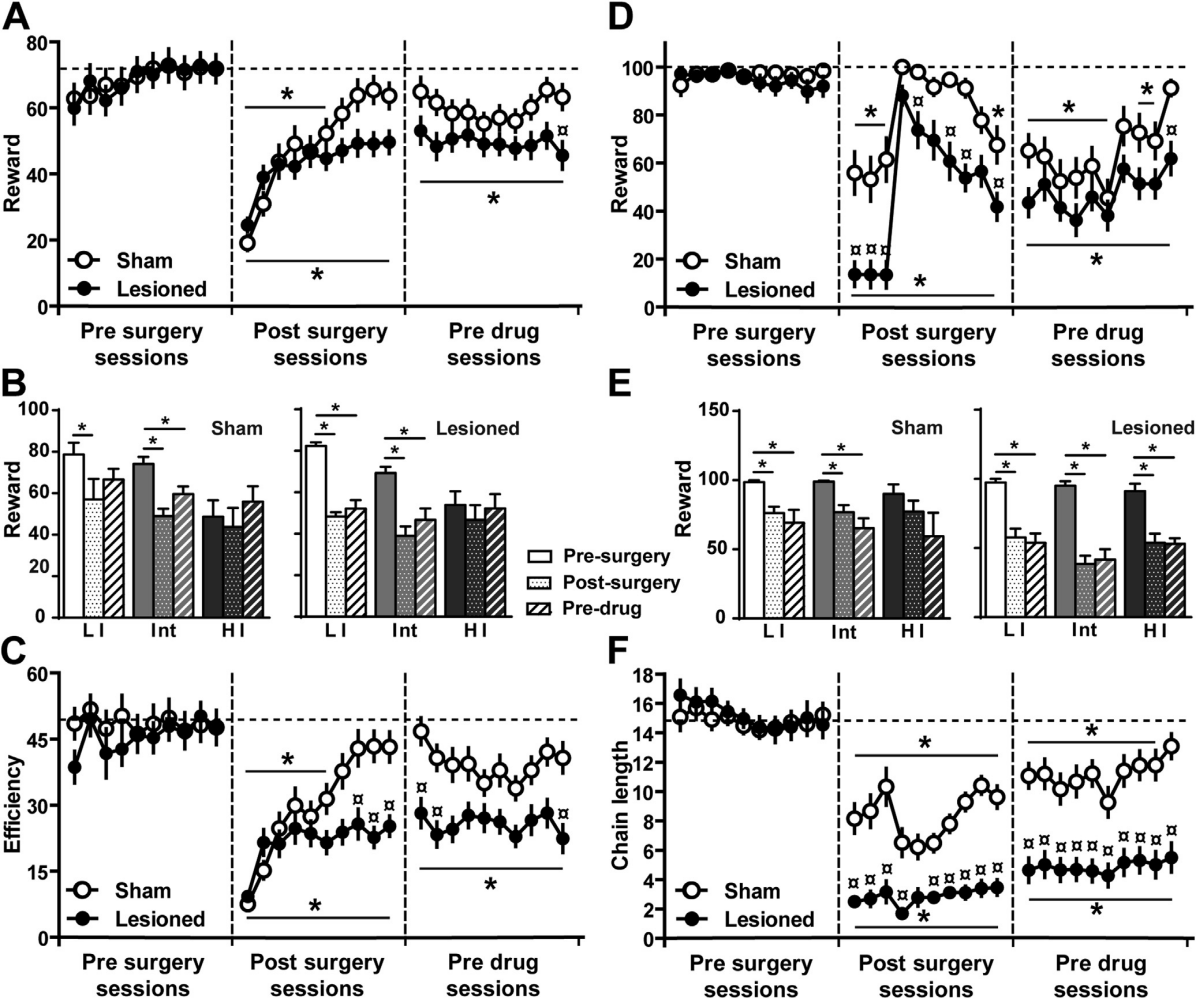
*Nigral dopaminergic lesion decreases inhibitory control*. **A**. Rewards obtained in DRL20 in baseline (pre surgery), for the 10 first sessions after surgery (post surgery) and for the 10 last sessions before drug challenge (pre drug) (all impulsivity traits included; 2-way ANOVA); **B**. DRL performances before surgery, after surgery and before drug treatment according to pre-existing impulsivity trait; **C**. Efficiency (rewards/responses) in DRL20 before surgery, after surgery and before drug challenge (2-way ANOVA); **D**. Rewards obtained in FCN16 before surgery, after surgery and before drug challenge (all impulsivity traits included; 2-way ANOVA); **E**. FCN performances before surgery, after surgery and before drug treatment according to pre-existing impulsivity trait; **F**. Chain length in FCN16 at the 3 periods (3-way ANOVA). Data represent mean ± SEM. p < 0.05 *from baseline, ¤ from sham, Sham: LI: n = 5, Int: n = 12, HI: n = 4; Lesioned: LI: n = 6, Int: n = 11, HI: n = 6.

Successful trials in the DRL task were influenced by impulsivity trait and the lesion (Lesion × Session: F_29, 1218_ = 2.21; p < 0.0001, Block × Impulsivity: F_4, 76_ = 7.86; p < 0.001 (three blocks of 10 sessions: pre-surgery, post-surgery and pre-drug)). Sham rats displayed a decrease in the number of rewards obtained over the first 6 sessions post-surgery (p < 0.05) but resumed their pre-surgery levels by session 8 (Fig. 2A). LI and Int rats were initially more affected than HI animals post-surgery (p < 0.05) and Int rats never recovered their pre-surgery performances (p < 0.05; Fig. 2B). While lesioned animals showed a similar alteration of waiting impulsivity, most of them never recovered their pre-surgery performance (p < 0.05) and performed worse than sham animals (last pre-drug session, p < 0.05; Fig. 2B). Interestingly, only HI rats were able to recover their pre-surgery performance (Fig. 2B). Similar results were observed for efficiency (Lesion × Session: F_29, 1218_ = 3.28; p < 0.0001). Sham rats exhibited a transient decrease in performance during the first 6 post-surgery sessions (p < 0.05), returning subsequently to their pre-surgery level (Fig. 2C). In marked contrast, lesioned rats remained impaired post-surgery (p < 0.05 from the last pre-surgery session) with an efficiency significantly lower than sham animals at post-surgery sessions 8–10 and pre-drug sessions 1, 2, and 10 (p < 0.05 for each). Efficiency on the last session before pre-surgery, post-surgery and before drug treatment (pre-drug) according to pre-existing impulsivity trait is shown on Suppl Fig. 3.

Rewards obtained in the FCN task were also affected by the lesion (Lesion × Session: F_29, 1218_ = 4.35; p < 0.0001) and impulsivity trait (Block × Sessions × Impulsivity: F_44, 763_ = 1.95 p < 0.0001). The number of rewards earned was decreased in sham animals for the first 3 post-surgery sessions for FCN16 (p < 0.05). When the FCN schedule was tailored for each animal to reach the baseline schedule (FCN16), performances progressively increased and sham rats recovered their pre-surgery scores by the last pre-drug session (Fig. 2D). In marked contrast, lesioned rats never recovered their pre-surgery level (p < 0.05), performing consistently worse than sham rats (p < 0.05, Fig. 2E). Regardless of impulsivity trait, all lesioned rats displayed a long-term deficit following surgery (p < 0.05 for both post-surgery and pre-drug periods; Fig. 2E). Consequently, FCN chain length was affected by both the lesion (Lesion × Session: F_29, 1218_ = 12.11; p < 0.0001) and impulsivity trait (Block × Sessions × Impulsivity: F_44, 396_ = 2.31; p < 0.0001). In sham animals, performances initially lower than pre-surgery (p < 0.05) recovered (Fig. 2F). In lesioned animals, chain length remained significantly lower from both baseline and sham animals in all post-surgery sessions (p < 0.05, Fig. 2F). Chain length on the last session before pre-surgery, post-surgery and before drug treatment (pre-drug) according to pre-existing impulsivity trait is shown on Suppl Fig. 3. During post-surgery, 35% of lesioned rats were under FCN3 compared with 5% of sham animals (p < 0.05), while 76% of sham rats were under FCN8 compared with 43% of lesioned rats (p < 0.05). Similarly, during the pre-drug period, only 26% of lesioned rats achieved the baseline criterion of FCN16 compared with 76% of sham rats (p < 0.05). This differential profile of performance in the FCN task over the course of the experiment is represented in Suppl Fig. 4 as the representativity of the sham and lesioned populations across the different FCN stages before surgery, after surgery and before drug challenge.

**Fig. 3:**
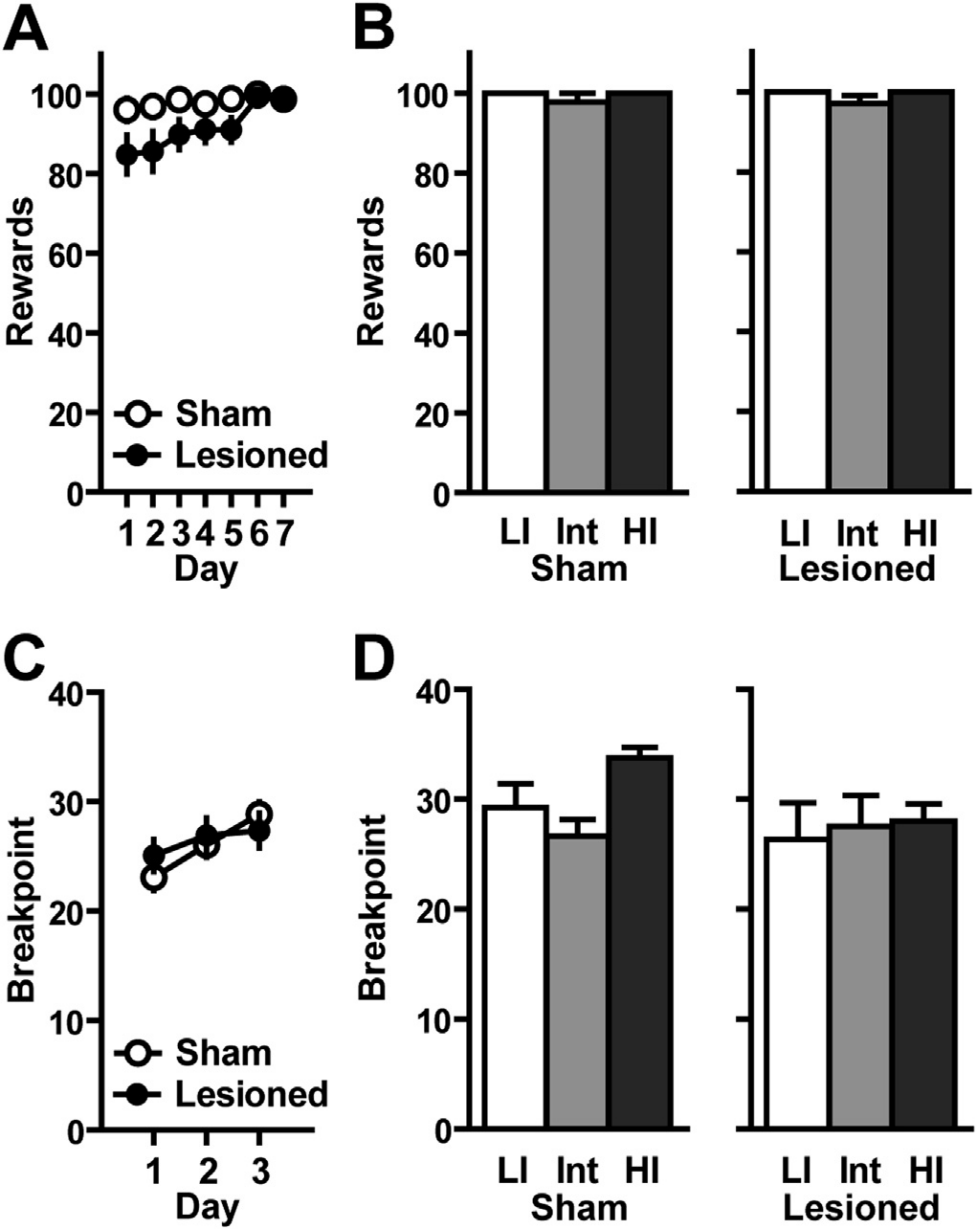
*Effects of bilateral SNc lesion on task acquisition*, *operant responding and motivation*: **A**. Over the 7 sessions, no difference was observed between sham and lesioned rats (all impulsivity traits included; 2-way RM-ANOVA) **B**. No difference in the total number of rewards obtained was seen between subpopulations of both groups for the last FR1 session (2-way ANOVA). **C**. The number of responses required to obtain a pellet increased according to the following progression (1, 3, 4, 6, 8, 11, 13, 16, 19, 23, 27, 32, 38, 44, 50, 58, 67, 77, 88, 100). No difference in breakpoint (the maximum number of wheel turn) was reported between both groups (along the 3 PR sessions (all impulsivity traits included; 2-way RM-ANOVA). **D**. No difference in breakpoints was seen between subpopulations of both sham and lesioned groups for the last PR session (2-way ANOVA). Sham: LI: n = 5, Int: n = 12, HI: n = 4; Lesioned: LI: n = 6, Int: n = 11, HI: n = 6.

Altogether, the long-term deficits displayed by lesioned rats illustrate the exacerbation by a nigral lesion of waiting impulsivity and impulsive actions. Importantly, such deficits were not attributable to motor, instrumental or motivational deficits. Indeed, to exclude a possible confounding effect of motor, motivational or instrumental conditioning impairments or motivation, rats were trained in a new task (wheel-turning) under both continuous (FR1) and progressive ratio (PR) schedules of reinforcement (Fig. 3). Neither lesion (Fig. 3A) nor impulsivity levels (Fig. 3B) influenced the acquisition of wheel-turning behavior over 7 days. Similarly, no effect of lesion (Fig. 3C) or impulsivity levels (Fig. 3D) were observed on breakpoints in the PR task.

### 3.3 Effects of dopaminergic treatments on waiting impulsivity

**Fig. 4:**
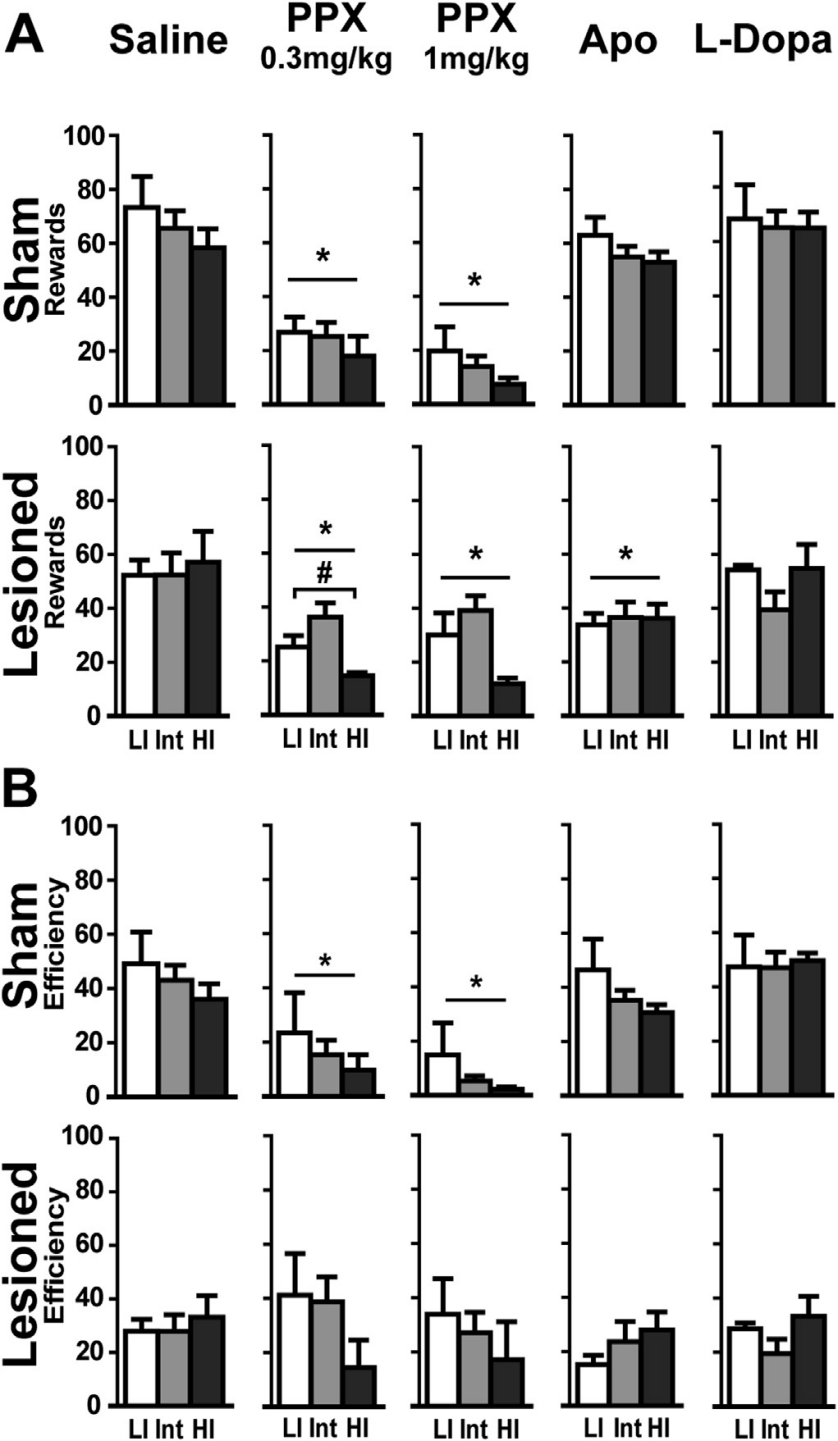
*Pramipexole differentially increases waiting impulsivity depending on SNc lesion and impulsivity trait*. **A**. Rewards obtained in DRL20 according to the dopaminergic drug; **B**. Efficiency (reward/response) in DRL20 during drug challenge. Data represent mean ± SEM; 2-way ANOVAs; *p < 0.01 from saline, # from Low impulsive. LI: Low impulsive, Int: Intermediate, HI: High impulsive; PPX: Pramipexole, Apo: Apomorphine. Sham: LI: n = 5, Int: n = 12, HI: n = 4; Lesioned: LI: n = 6, Int: n = 11, HI: n = 6.

Impulse control in the DRL task was impaired by dopamine receptor agonists but not by L-Dopa (lesion × Treatment: F_4, 210_ = 8.35; p < 0.001) (Fig. 4). The number of rewards collected was decreased by apomorphine in all lesioned rats (p < 0.001 vs. saline) and by PPX, at both doses in all rats (ps < 0.001 vs. saline). While the effect of PPX treatment was not dependent upon pre-existing impulsivity trait in sham rats, a trend in lesioned animals (Treatment × Impulsivity: F_8, 80_ = 1.80; p = 0.08) suggested potential differences between HI, Int and LI rats. ANOVAs with planed comparisons carried out on LI vs. HI rats revealed that HI rats under 0.3 mg/kg PPX received less rewards (F_1, 20_ = 4.50; p < 0.05; Fig. 4A).

Sham animals presented a decreased efficiency (Treatment × lesion: F_4, 210_ = 10.03; p < 0.0001) following both PPX doses (ps < 0.01, Fig. 4B). Despite a decrease in rewards obtained and an increase in reward-seeking comparable to sham animals (67.35 ± 10.48 vs. 46.77 ± 8.65 tray nosepokes/rewards for PPX 1 mg/kg; Treatment × lesion: F_4, 210_ = 3.16; p < 0.05), lesioned animals did not display an altered efficiency.

**Fig. 5:**
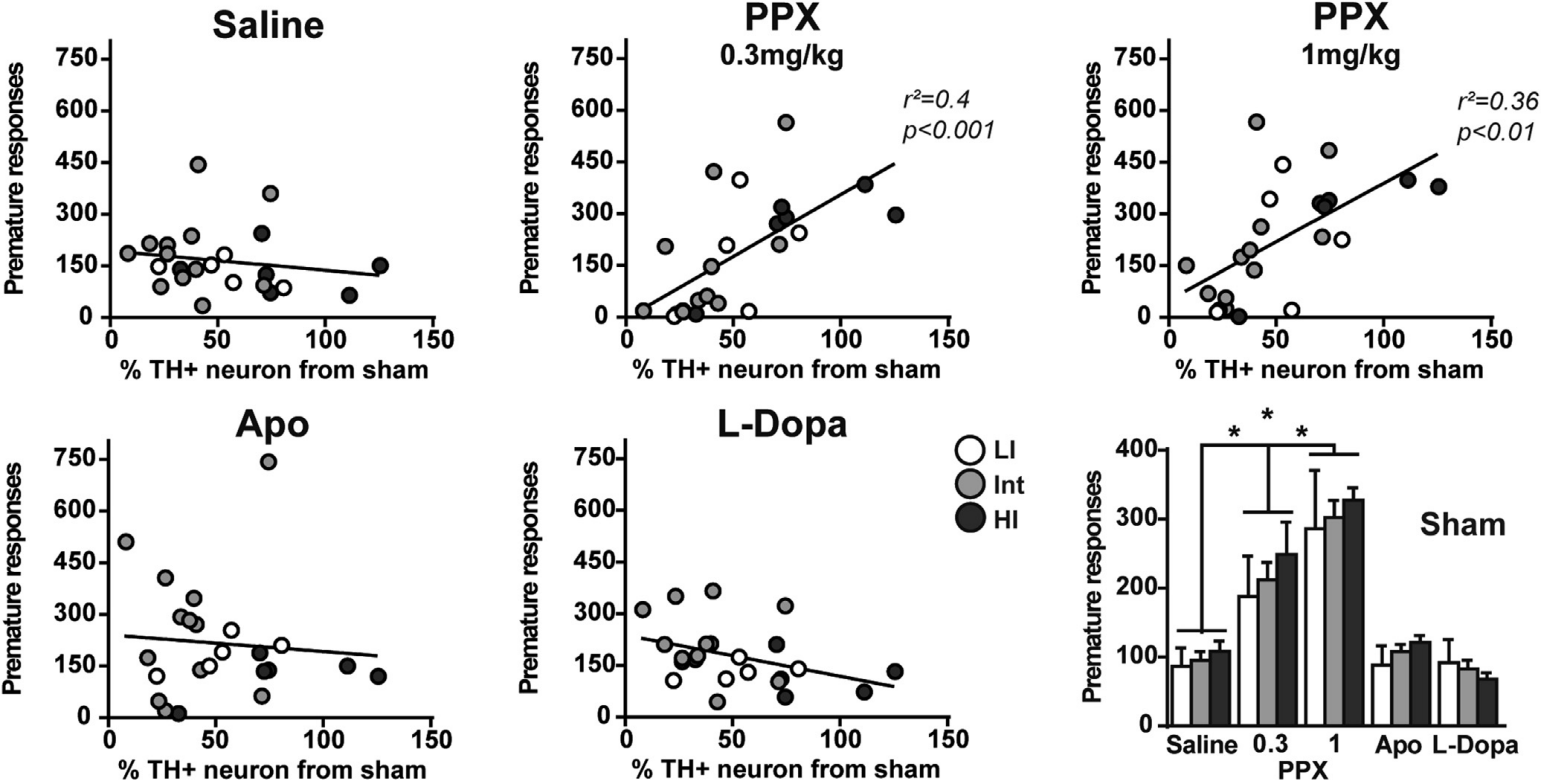
*Premature responses under PPX are dependent on impulsivity and lesion severity*. Linear regressions represent premature responses according to lesion compared to sham rats; Lower right panel represent the number of premature responses for sham rats according to the different dopaminergic drugs (2-way ANOVA). Data represent mean ± SEM; *p < 0.0001. LI: Low impulsive, Int: Intermediate, HI: High impulsive; PPX: Pramipexole, Apo: Apomorphine; TH+: Tyrosine Hydroxylase positive. Sham: LI: n = 5, Int: n = 12, HI: n = 4; Lesioned: LI: n = 6, Int: n = 11, HI: n = 6.

Linear regressions revealed that animals with lower lesion levels made more premature responses under both PPX doses (r^2^ = 0.4, p < 0.001 and r^2^ = 0.36, p < 0.01 respectively; Fig. 5). Hence, despite a milder loss of dopaminergic neurons, HI rats were more sensitive to the disinhibitory effects of PPX than LI rats. In sham rats, premature responses increased dose-dependently with PPX regardless of impulsive trait (Treatment: F_4, 90_ = 32.63; p < 0.0001, Treatment × impulsivity interaction: F_8, 90_ < 1; Fig. 5).

### 3.4 Effects of dopaminergic treatments on impulsive actions

**Fig. 6:**
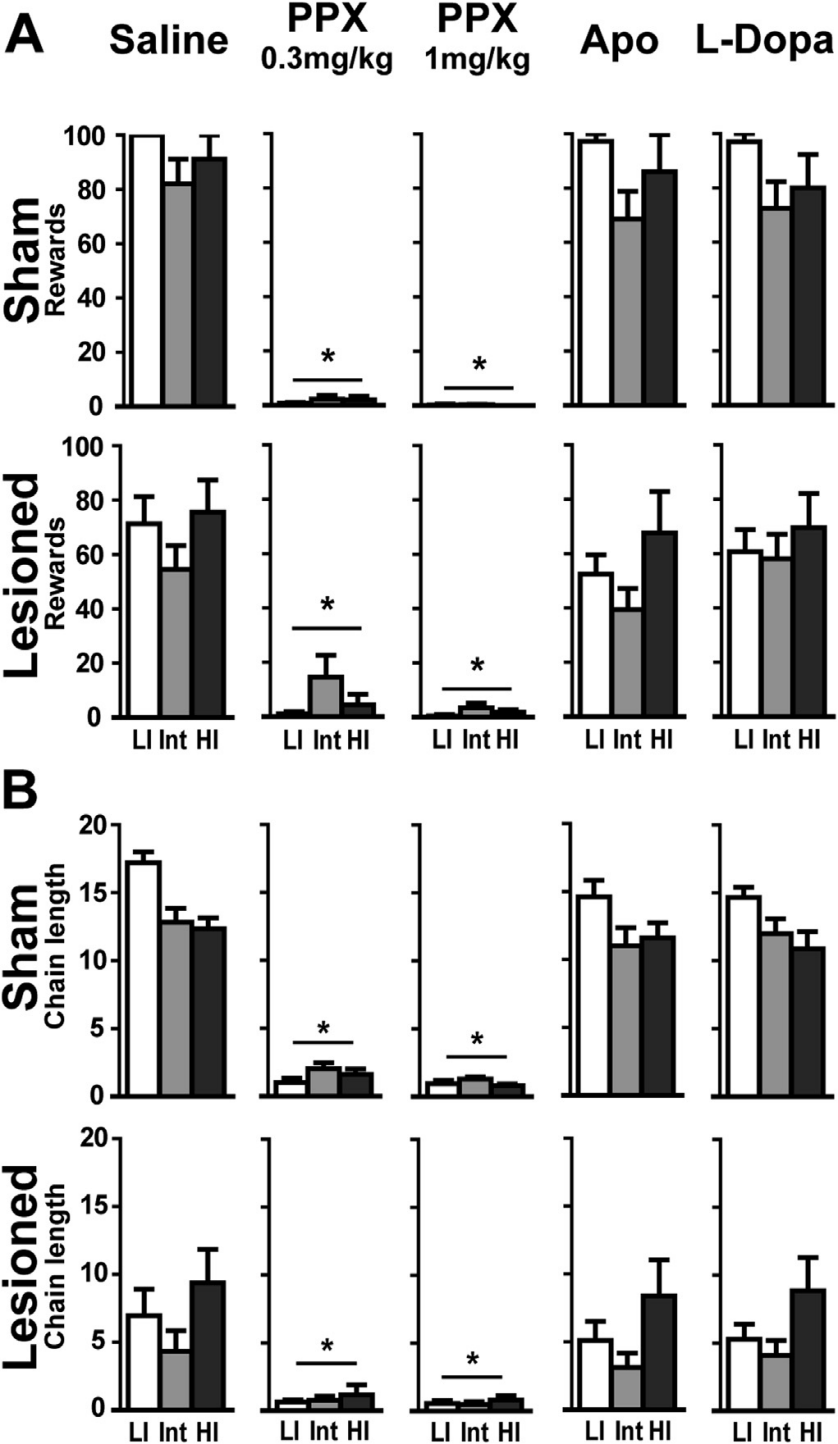
Pramipexole *increases impulsive action regardless of SNc lesion or impulsivity trait*. **A**. Rewards obtained in FCN according to the dopaminergic drugs; **B**. Chain length in FCN during drug challenge. Data represent mean ± SEM; 2-way ANOVAs; *p < 0.001 from saline. LI: Low impulsive, Int: Intermediate, HI: High impulsive; PPX: Pramipexole, Apo: Apomorphine. Sham: LI: n = 5, Int: n = 12, HI: n = 4; Lesioned: LI: n = 6, Int: n = 11, HI: n = 6.

In the FCN test, the number of rewards collected and chain length were reduced by PPX (Lesion × Treatment: F_4, 210_ = 4.84; p < 0.001 and F_4, 210_ = 10.79; p < 0.001, respectively), but not by apomorphine or L-Dopa (Fig. 6). Sham and lesioned rats displayed a drastic decrease in the number of rewards obtained and chain length with both PPX doses (ps < 0.001 vs saline, Fig. 6A and B, respectively) the extent of which preventing investigating any influence of impulsivity. Indeed, 43% or 76% of sham and 43.5% or 52% of lesioned rats obtained no reward after injection of 0.3 or 1 mg/kg PPX, respectively (ps < 0.01 vs. saline). Neither apomorphine nor L-Dopa altered FCN performances in sham and lesioned rats.

## 4 Discussion

Impulsivity has been described in PD (Dagher and Robbins, 2009) but the underlying pathophysiological substrates and their etiological contribution to ICDs remain poorly known (Poletti and Bonuccelli, 2012). Waiting impulsivity and impulsive actions (D’Amour-Horvat and Leyton, 2014) are well documented in neurologically intact subjects and are modulated by dopamine, particularly with the mesolimbic and mesocortical pathways. In PD, dopaminergic function is altered by the nigrostriatal dopaminergic loss but also by DRT, and clinical studies have provided opposite findings on the contribution of these factors to altered inhibitory control (Bastiaens et al., 2013, Vriend et al., 2015, Weintraub et al., 2013). Besides methodological differences, these discrepancies may stem from the multifactorial etiology of ICDs and the heterogeneity of the disease process (Nombela et al., 2014). This longitudinal study was therefore designed to disentangle the respective contributions of nigrostriatal neurodegeneration, DRT and baseline impulsivity in the emergence of behavioral deficits relevant to ICDs.

Our results provide the first evidence that impulsivity is associated with a differential vulnerability to α-synuclein-induced dopaminergic neurodegeneration, with HI rats being less sensitive than Int and LI rats. This is in agreement with the observation that impulsivity is decreased in mice lacking α-synuclein (Pena-Oliver et al., 2012, Pena-Oliver et al., 2014), suggesting that highly impulsive subjects can cope with higher levels of α-synuclein that non impulsive. Impulsivity is associated with decreased midbrain D2/D3 autoreceptor availability and increased amphetamine-induced striatal dopamine release (Buckholtz et al., 2010), suggesting that in impulsive individuals dopamine neurons may be able to sustain a higher activity, metabolic demand and proteostasis, which may overall help them to cope more efficiently with alpha-synuclein overexpression.

The present results further demonstrate that nigrostriatal degeneration affects several dimensions of impulsivity. Lesioned animals displayed a major decline in DRL efficiency indicating increased waiting impulsivity. However, performances of LI and Int lesioned rats were more affected by the lesion than those of HI rats, a difference potentially attributable to the milder nigrostriatal degeneration observed in the latter. The nigrostriatal lesion also drastically increased impulsive actions. Even though the differences in DRL performances between impulsivity subgroups could be related to a different magnitude of nigral degeneration, the similar FCN impairment between HI and LI lesioned rats despite a milder dopaminergic loss in HI rats suggest that impulsive individuals may be more sensitive to the deleterious effect of dopaminergic loss on impulsive actions. These results also suggest that mesencephalic dopaminergic nuclei may differentially contribute to impulsive actions depending on impulsive trait.

Collectively, our results indicate that a neurodegenerative process affecting the nigrostriatal pathway in rats, akin to the neurodegeneration observed in PD patients, increases independent dimensions of impulsivity (waiting impulsivity and impulsive actions). This deleterious effect of the nigrostriatal lesion on impulse control seemed to be more pronounced on impulsive actions than on waiting impulsivity, particularly regarding HI lesioned rats. Our results also suggest that premorbid impulsivity may interact with the disease process in PD and differentially influence the nature of the inhibitory control deficits developing after nigrostriatal degeneration. Nigrostriatal degeneration can also affect other dimensions of impulsivity, as recently shown with increased impulsive choice in a delay discounting task following dorsolateral striatal dopaminergic loss (Tedford et al., 2015). While decreased inhibitory control may not be sufficient *per se* to trigger ICDs in PD patients, our results suggest that dopaminergic neurodegeneration can affect behavioral traits to a much greater extent than previously thought (Bastiaens et al., 2013, Weintraub et al., 2013).

Dopamine depletion can also decrease willingness to sustain the effort to obtain rewards (Cawley et al., 2013), a motivational deficit that cannot account for the observed results since lesioned rats did not display instrumental or motivational deficits as assessed using a separate series of food-reinforced instrumental tasks.

We further evidenced that the influence of DRT over waiting impulsivity depends on impulsivity trait in lesioned rats only. Consistent with clinical studies that identified D2/D3 agonists as a major risk factor for ICD (Voon et al., 2006, Weintraub, 2009), the D2/D3 agonist PPX was the most deleterious DRT, increasing waiting impulsivity in both sham and lesioned groups. While all sham rats were equally susceptible to this deleterious effect, a different pattern was found in lesioned rats. Indeed, HI lesioned rats that were the only lesioned animals able to recover their pre-lesion performance in the DRL task, obtained less rewards than their LI counterparts when treated with the lowest PPX dose. These results suggest that the lesion, DRT and impulsive trait can interact to further increase waiting impulsivity in high impulsive individuals. Surprisingly, efficiency was not significantly reduced by DRT in lesioned rats albeit they expressed a similar reward-seeking behavior as sham rats. Efficiency as a measure of impulsivity can however carry limitations, as unfocused behaviors could mask poor inhibitory capacities (Hill et al., 2012, Sanabria and Killeen, 2008). Further highlighting multiple contributions to the emergence of greater inhibitory control deficits, premature responses under PPX in the DRL task negatively correlated with lesion severity, with HI animals displaying more premature responses being those with the mildest lesion.

The non-selective dopamine receptor agonist apomorphine increased impulsive actions in all lesioned rats. Hypokinesia in PD is known to partly rely on D1 receptor signaling pathway hypoactivation (Mallet et al., 2006). The activation of this pathway by apomorphine may have resulted in a sudden increased activity leading to poor DRL performances. Such result is consistent with previous work showing that both D1 and D2 receptors stimulation mediate behavioral disinhibition and premature responding in the 5-choice serial reaction time task (van Gaalen et al., 2006).

While studies using discounting tasks in human showed that impulsive choices were related to D2/3 agonists exclusively in patients presenting ICDs (Voon et al., 2010b), our results show that PPX drastically increased impulsive actions independently of a dopaminergic lesion or impulsivity trait. Since impulsive actions depend heavily upon dopaminergic signaling in mesolimbic pathways (Basar et al., 2010) that were spared both in normal and lesioned rats, a dopamine overdose of fronto-limbic networks following PPX administration may underlie this effect. Nonetheless, these results show that D2/3 agonists trigger impulsive behaviors in both SNc lesioned and non-lesioned subjects as evidenced in PD (Weintraub et al., 2010) and the restless legs syndrome (Voon et al., 2011c).

Importantly, our results showing that PPX differentially alters impulsive actions and waiting impulsivity are in agreement with a recent study demonstrating that dimensions of impulsivity are not equally affected by PPX in PD patients (Antonelli et al., 2014). This effect of PPX is also in line with clinical and neurocomputational results demonstrating that enhanced D2 stimulation favors a “Go” bias and decreases the ability to suppress inappropriate responses (“NoGo”) (Frank et al., 2004). Although the enhancement of impulsivity reported here may represent a core feature underlying the propensity of D2/D3 agonists to induce ICDs, PPX also alters learning from outcomes (Piray et al., 2014, Voon et al., 2010a) and biases choices towards risky options both in rats (Johnson et al., 2011, Rokosik and Napier, 2012) and PD patients (Voon et al., 2011a), thus disrupting cognitive processes that are necessary for optimal decision making and altered in patients with ICDs.

## 5 Conclusions

This study in a bilateral rat model of PD shows that nigrostriatal dopaminergic neurodegeneration, DRT and pre-morbid impulsivity interact, and differentially contribute to alter inhibitory control. Nigrostriatal neurodegeneration increases impulsivity but waiting impulsivity and impulsive actions are differentially affected depending on baseline impulsivity trait. Indeed, α-synuclein overexpression increases impulsive actions but not waiting impulsivity in individuals with high basal impulsivity but impairs global inhibitory control in rats with low basal impulsivity. Despite overexpression of α-synuclein, HI animals present a reduced nigrostriatal degeneration compared to LI rats, supporting the hypothesis that behavioral traits can be associated with a differential vulnerability of dopaminergic neurons to degeneration. In addition to establishing the face validity and clinical relevance of this model to study the pathophysiology of impulsivity and of non-motor side effects of DRT in PD, we show that if impulsive actions are highly sensitive to PPX regardless of a dopaminergic loss or impulsivity trait, PPX-induced increase in waiting impulsivity depends upon the combination of nigral degeneration and pre-existing impulsivity trait.
