# Supplementary material for: Multi-facetted impulsivity following nigral degeneration and dopamine replacement therapy
Source: Neuropharmacology. 2016 Oct;109:69–77. doi: 10.1016/j.neuropharm.2016.05.013 (PMC5405054; doi:10.1016/j.neuropharm.2016.05.013)
Supplement: Supplementary file 1 [file mmc1.docx]

**Supplementary material**


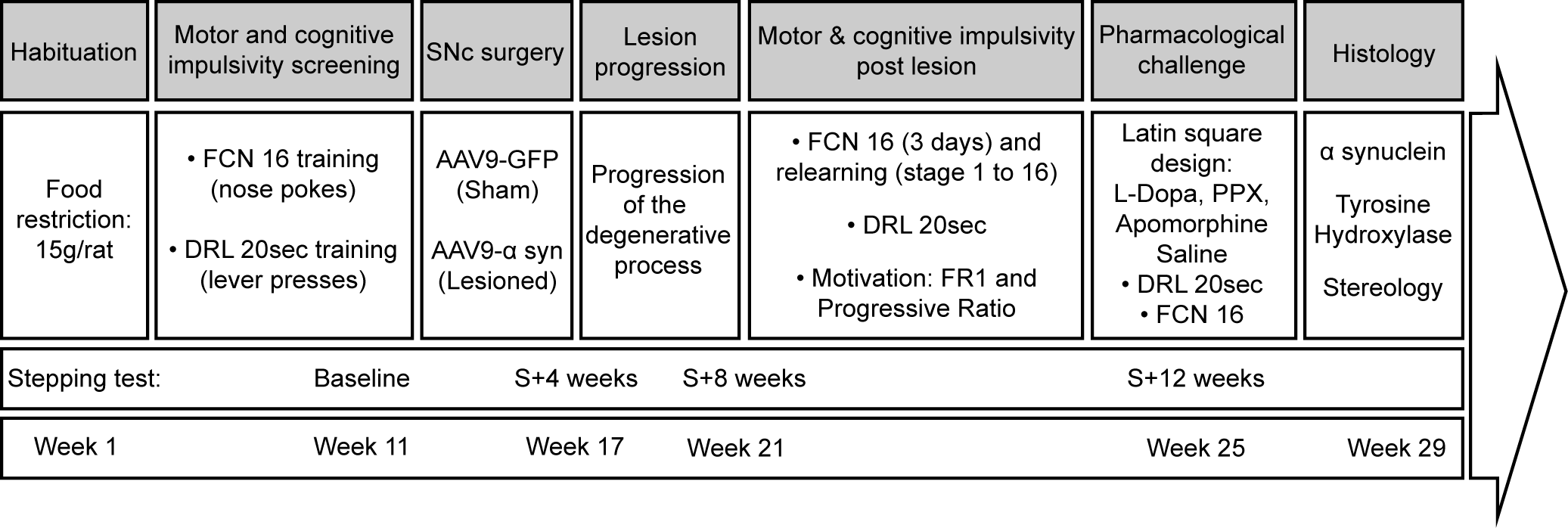


**Supplementary Figure 1**: *Experimental design*.

Food restricted animals were trained for DRL and FCN tasks in order to screen Low Impulsive (LI), Intermediate (Int) and High Impulsive (HI) subpopulations. Both tasks were performed using different manipulanda and training sequences were randomly assigned to avoid any carry-over effect. Bilateral lesion of the substantia nigra *pars compacta* was achieved via viral-mediated bilateral overexpression of α-synuclein*.* 8 weeks following the surgery rats were re-trained and their behavioural performance in both tasks was assessed for 20 sessions, 10 considered to reflect the reacquisition of the performance in the task (post-surgery period) and the following 10 their baseline performance prior to dopaminergic treatments (pre-drug period). Finally, brains were processed for α-synuclein and tyrosine-hydroxylase immunohistochemistry.


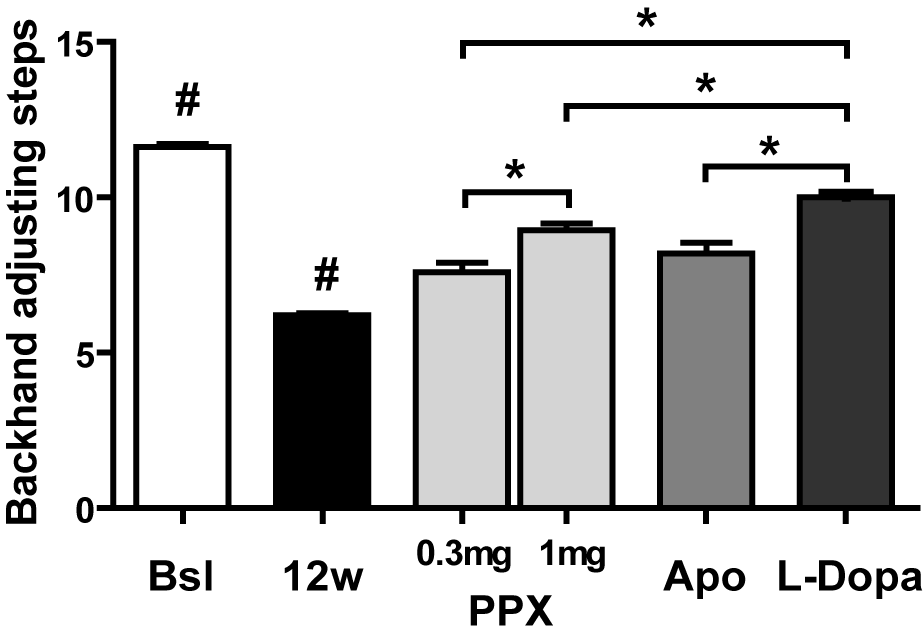


**Supplementary Figure 2**: Effects of DRT on motor impairments.

Motor impairments were improved by all dopaminergic drug tested (one-way ANOVA: F_5,73_ = 298.8; p < .0001). Newman-Keuls post-hoc analyses highlighted motor improvement for both PPX doses (ps < .001) with more pronounced effects of the 1mg/kg dose (p < .001 vs 0.3 mg/kg). Apomorphine also improved stepping performances (p < .001). L-Dopa was the most effective drug (p < .001 vs all drugs). Each drug was tested on representative samples of 5-8 animals containing LI, Int and HI individuals. Bsl: Baseline; 12w: 12 weeks; PPX: Pramipexole; Apo: apomorphine. * p < .001, # p < .001 vs all drugs. All data are mean ± SEM.


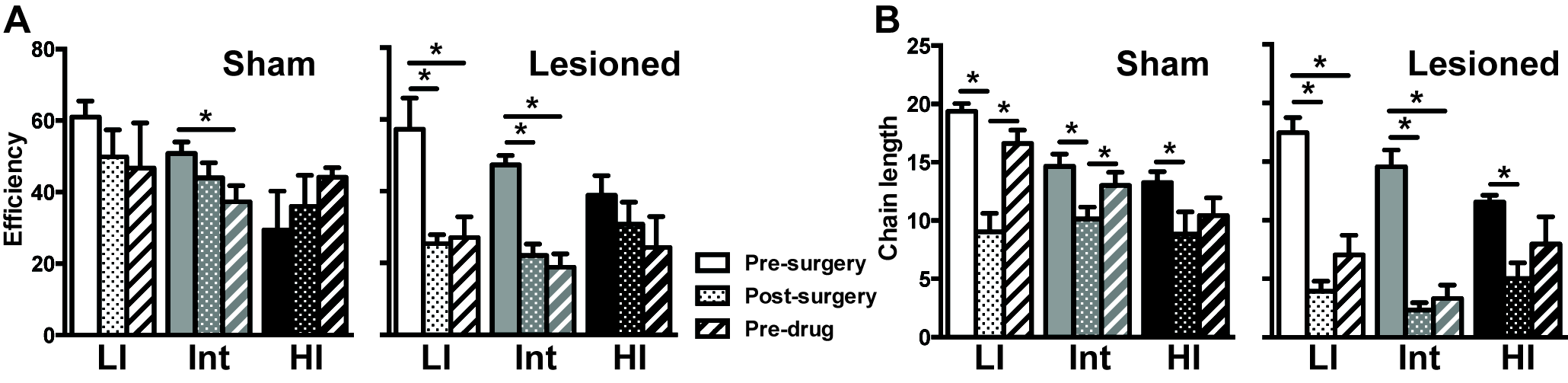


**Supplementary Figure 3 :** effect of the nigrostriatal lesion and impulsivity trait on inhibitory control

**A.** Efficiency (rewards/responses) in DRL20 on the last session pre-surgery, post-surgery and before drug treatment (pre-drug) according to pre-existing impulsivity trait. **B.** Chain length in FCN16 on the last session pre-surgery, post-surgery and before drug treatment (pre-drug) according to pre-existing impulsivity trait. * p < .05. Sham: LI: n=5, Int: n=12, HI: n=4; Lesioned: LI: n=6, Int: n=11, HI: n=6.


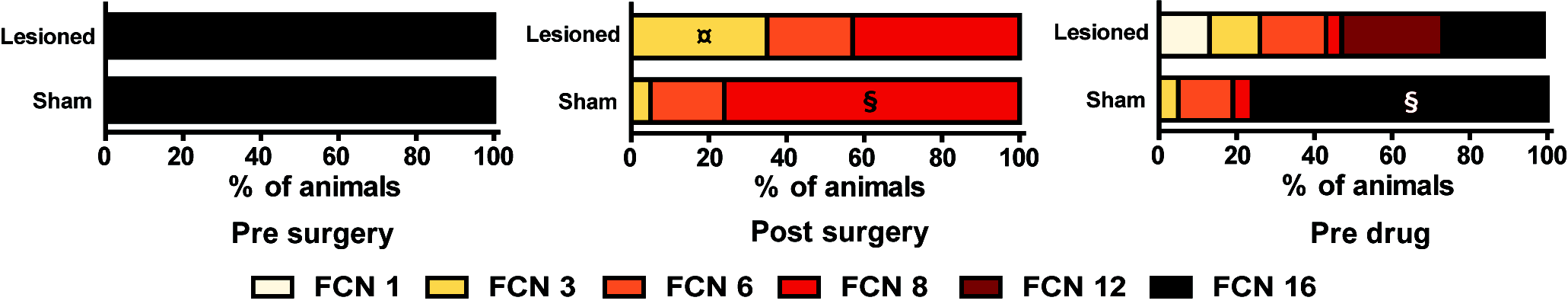


**Supplementary Figure 4**: Effect of the nigrostriatal lesion on FCN re-acquisition

Distribution of the sham and lesioned populations across the FCN stages before surgery, after surgery and before drug challenge. Data represent mean ± SEM; p < .05 ¤ from sham, § from lesioned (Chi²). Sham: LI: n=5, Int: n=12, HI: n=4; Lesioned: LI: n=6, Int: n=11, HI: n=6.
